# The *RGS2* (-391, C>G) Genetic Variation Correlates to Antihypertensive Drug Responses in Chinese Patients with Essential Hypertension

**DOI:** 10.1371/journal.pone.0121483

**Published:** 2015-04-07

**Authors:** Fazhong He, Jianquan Luo, Zhitao Zhang, Zhiying Luo, Lan Fan, Yijing He, Jiagen Wen, Dingilang Zhu, Jinping Gao, Yan Wang, Yuesheng Qian, Honghao Zhou, Xiaoping Chen, Wei Zhang

**Affiliations:** 1 Department of Clinical Pharmacology, Xiangya Hospital, Central South University, Changsha, P.R.C; 2 Shanghai Institute of Hypertension, Ruijin Hospital, Shanghai Jiaotong University School of Medicine, Shanghai, P.R.C; 3 Second uropoiesis surgical department in Han Dan Central Hospital, Handan, P.R.C; 4 Institute of Clinical Pharmacology, Central South University, Changsha, P.R.C; 5 Hunan Key Laboratory of Pharmacogenetics, Changsha, P.R.C; IPO, Portuguese Institute of Oncology of Porto, PORTUGAL

## Abstract

**Objective:**

Regulators of G-protein signaling protein 2 (*RGS2*) play an irreplaceable role in the control of normal blood pressure (BP). One *RGS2* (-391, C>G) genetic variation markedly changes its mRNA expression levels. This study explored the relationship between this genetic variation and the responses to antihypertensive drugs in Chinese patients with essential hypertension.

**Methods:**

Genetic variations of *RGS2* were successfully identified in 367 specimens using polymerase chain reaction-restriction fragment length polymorphism (PCR-RFLP) assays. All patients were treated with conventional doses of antihypertensives after a 2-week run-in period and followed-up according to our protocol. A general linear model multivariate analysis of variance (ANOVA) was used for the data analysis.

**Results:**

A significant difference in the mean systolic BP change was observed between *RGS2* (-391, C>G) CC/CG (n = 82) and GG (n = 38) genotype carriers (-13.6 vs. -19.9 mmHg, P = 0.043) who were treated with candesartan, irbesartan or imidapril at the end of 6 weeks. In addition, the patients’ BP responses to α,β-adrenergic receptor blockers exhibited an age-specific association with the *RGS2* (-391, C>G) genetic variation at the end of 4 weeks.

**Conclusion:**

The *RGS2* (-391, C>G) genetic polymorphism may serve as a biomarker to predict a patient’s response to antihypertensive drug therapy, but future studies need to confirm this.

## Introduction

Regulator of G-protein signaling protein 2 (*RGS2*), also known as G0S8, belongs to a set of putative G0/G1 switch regulatory genes that are selective and highly regulated inhibitors of G_q/11_ signaling, and they play critical roles in regulating vascular tone and cardiac remodeling [1,2,3]. Previous studies revealed that mice with global *RGS2* deletion have a hypertensive phenotype, which was initially attributed to enhanced and prolonged G_q/11_-mediated vascular pressor responses and impaired nitric oxide (NO)-induced, cGMP-mediated relaxation responses [4,5]. Clinical studies observed significant decreases in *RGS2* mRNA and protein levels in peripheral blood mononuclear cells (PBMs) and fibroblasts from hypertensive patients compared to normotensive subjects. The intracellular Ca^2+^ peak elicited by Ang II was greater in the fibroblasts from hypertensive patients compared to those from normotensive subjects, and the *RGS2* mRNA levels were negatively correlated [2]. Another study demonstrated that alterations in *RGS2* expression levels or functionality were implicated in the deregulation of Ang II signaling and abnormal aldosterone secretion by the adrenal gland [6]. Semplicini et al. indicated that low *RGS2* expression is the leading cause of antihypertensive drug resistance because of poor negative feedback on the effects of aldosterone and other vasoactive agents [7].

Recent studies reported that *RGS2* mRNA levels were also significantly up-regulated in response to an α1-adrenergic receptor (ADR) blocker and β-ADR agonist [8,9,10]. *RGS2* also selectively terminates α1-ADR agonist-induced Gq/11-specific and β-ADR agonist-mediated Gi signaling pathways [11,12]. In addition, telmisartan significantly improved the survival rates and suppressed vascular remodeling in an *RGS2*-deficient mice model. Other studies suggest that Ang II causes a rapid and transient increase in *RGS2* mRNA levels, which can be blocked by the calcium channel blocker (CCB) nifedipine[6,13]. Notably, one recent study observed that a cAMP-response element played a critical role in activating the *RGS2* promoter, and the -867 to -367 bp region had an integral role in basal promoter activation [14]. Further research indicated that *RGS2* mRNA levels were up-regulated because of an *RGS2* (-391, C>G) mutation [15], and this mutation was highly frequent in population distributions [16]. *RGS2* (-218, C>G, -204, G>T, -203, C>T) genetic variations abolished Ang II- and forskolin-induced *RGS2* mRNA overexpression. However, these mutations are highly conserved, and the allele frequencies were < 2% [17]. Therefore, we hypothesize that the *RGS2* (-391, C>G) genetic variation plays an important role in regulating blood pressure and the response to antihypertensive drug therapy.

## Methods

### Subjects

The study protocol was described in detail in a previous paper [18], but the key elements are summarized below. The Ethics Committee of Xiangya School of Medicine at Central South University approved the study protocol, and the registration number (ChiCTR-RO-12002612) is validated on the Chinese Clinical Trial Registry website (http://www.chictr.org/cn/). For genetic screening, our study recruited a total of 367 subjects with primary mild to moderate essential hypertension who were 26 to 81 years of age. All subjects provided written informed consent. The patients were enrolled after a 2-week run-in period and were assigned to receive the drugs for 4 or more weeks. The patients were followed up every 2 or 4 weeks on the weekends. Details of the entire protocol, such as BP measurements and inclusion and exclusion criteria, are available in a previous paper [18].

### Genotyping procedure for *RGS2*

The *RGS2* (rs2746072, -391,C>G,) genotypes were determined using a polymerase chain reaction-restriction fragment length polymorphism (PCR-RFLP) assay. The genotyping procedure for the *RGS2* gene was performed as previously described, with minor modifications [19]. The final 25 μl PCR mixture contained the following components: a 10x PCR buffer (2.5 μl), 10x dNTP (2.5 μl), 10 μM of each of the forward and reverse primers (0.5 μl), H_2_O (16.8 μl), g—DNA (2 μl), and Taq-ase (0.2 μl). The temperature cycling program was initial denaturation for 5 minutes at 95.8°C, followed by 36 cycles of denaturation at 95.8°C for 30 s, annealing at 56.1°C for 30 s, and elongation at 72°C for 30s, and a terminal extension for 5 minutes at 72°C. Amplified *RGS2* DNA fragments were hydrolyzed by BsiEI at 37°C for 16 hours, and the hydrolysates were electrophoresed in ethidium bromide-stained 2.5% agarose gels and visualized using an electrophoresis apparatus.

### Statistical analysis

The patients’ characteristics are reported as means and standard deviation (SD) for continuous variables, and frequencies and percentages are reported for the categorical data. Deviations from Hardy-Weinberg equilibrium of single nucleotide polymorphisms (SNPs) were investigated using the χ2 test. Differences between the baseline characteristics of single SNPs and any given phenotype of interest were assessed using independent-sample T-tests or Wilcoxon rank-sum tests, as appropriate. In terms of the heart rate and blood pressure data, general linear model multivariate analysis of variance (ANOVA) was performed to compare the genotype groups with *RGS2* (-391,C>G) as a factor, and body mass index (BMI), age, sex were used as covariates. Stratified analysis and multivariate ANOVA were used to assess significant interactions between age-genotype and gender-genotype. A two-tailed P-value of <0.05 was considered to be significant. Data analysis was performed using SPSS, version 19.0 for Windows (IBM, Chicago, IL, USA).

## Results

In this study, 367 patients were unambiguously genotyped for the *RGS2* (-391, C>G) genetic variation. The genotype frequencies for *RGS2* (-391, C>G) GG, GC, and CC were 31.6%, 49.0%, and 19.4%, respectively, and the allele frequencies were in Hardy-Weinberg equilibrium (P>0.05). The G allele frequency was 43.8% in the Chinese population. The genotype baseline characteristics of the patients, as stratified by the *RGS2* genotype, are shown in Table 1. There were no between-group differences in age, BMI, HR, BP or other clinical characteristics (e.g., FBG, TG, CHO, HDL and LDL).

**Table 1:** Comparison of the baseline characteristics of the eligible patients in the *RGS2* (-391, C>G) genotype groups.

Variable	RGS2		P-value
	GG	GC+CC	
Sex(male/female)	67/49	142/109	
Age(years)	57.0±8.6(116)	56.8±8.4(251)	0.873
BMI(kg/m^2)^	24.7±3.7(116)	25.3±3.0(250)	0.097
HR(bpm)	75.4±7.8(116)	75.1±7.3(251)	0.980
SBP(mm Hg)	151.2±11.2(116)	149.4±10.0(251)	0.129
DBP(mm Hg)	98.2±4.6(116)	98.0±4.3(251)	0.465
MAP(mm Hg)	115.9±5.7(116)	115.1±5.3(251)	0.220
PP(mm Hg)	52.9±10.4(116)	51.4±9.0(251)	0.298
ALT(μmol/L)	30.4±15.3(99)	32.0±23.4(213)	0.790
BUN(mmol/L)	5.1±1.3(102)	5.8±6.5(228)	0.450
UCr(mmol/L)	78.2±16.1(113)	80.0±16.7(245)	0.337
UA(mmol/L)	320.5±83.0(73)	323.1±83.6(188)	0.821
FBG(mmol/L)	5.3±1.0(102)	5.3±1.1(212)	0.490
TG(mmol/)	1.8±2.0(112)	1.8±1.3(242)	0.880
CHO(mmol/L)	5.1±1.0(113)	5.2±1.2(244)	0.251
HDL(μmol/L)	1.4±0.3(104)	1.4±0.3(227)	0.650
LDL(μmol/L)	3.4±1.0(75)	3.6±1.1(147)	0.213

Data are expressed as means ± SD. BMI indicates body mass index; HR, heart rate; SBP, systolic blood pressure; DBP, diastolic blood pressure; PP, pulse pressure; MAP, mean arterial pressure; ALT, alanine aminotransferase; BUN, blood urea nitrogen, UCr, urine creatinine; UA, uric acid; FBG, fasting blood glucose; TG, triglyceride; CHO, cholesterol; HDL, high-density lipoprotein; and LDL, low-density lipoprotein.

**Fig 1:**
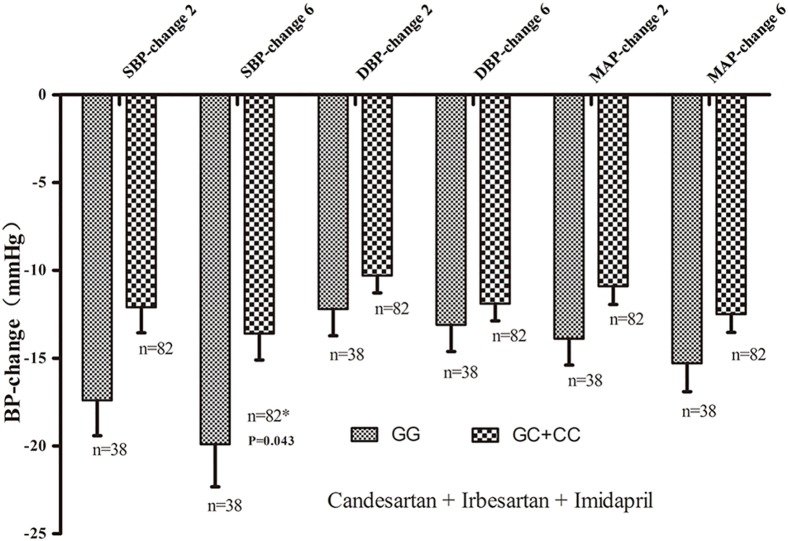
The relationship between the *RGS2* (-391, C>G) genetic polymorphism and the hypotensive effects of candesartan, irbesartan or imidapril monotherapy in EH patients. *P-values are shown with age, BMI, and gender adjustments. The error bar indicates 95% CI.

After adjusting for age, sex and BMI, a significant SBP difference was observed between *RGS2* (-391, C>G) GG and CC/CG genotype carriers in the candesartan, irbesartan or imidapril treatment groups at the end of 6 weeks (Fig 1). Ang-(1–7) regulates blood pressure by promoting pro-inflammatory and pro-thrombotic effects, and Ang IV can ameliorate AngII-induced cardiac injury via *AT4R* and protect against acute cerebral ischemia via Ang IV-AT4R and NO-dependent mechanisms [20,21]. We further analyzed the effects of the *RGS2* (-391, C>G) genetic variation between the angiotensin receptor blocker (ARB) treatment group and the ACE inhibitor treatment group. SBP changes were observed between the *RGS2* (-391, C>G) CC/CG and GG genotype carriers who were treated with candesartan or irbesartan therapy at the end of two and six weeks. Specific details of these differences are shown in Table 2. Imidapril did not have a different blood pressure-lowering effect in the different *RGS2* (-391, C>G) genotype groups.

**Table 2:** The blood pressure response to candesartan and irbesartan therapy, as stratified by the RGS 2 (-391, C >G) genotype.

**Variables**	**-391, C>G genotype of *RGS2*(n)**		* **P-value**
	GG (n = 17), 95% confidence interval (CI)	GC+CC (n = 32), 95% CI	
△SBP2, mmHg	-22.1(-27.5,-16.7)	-13.1(-17.1,-9.0)	**0.008**
△SBP4, mmHg	-16.6(-22.8,-10.3)	-14.3(-18.9,-9.7)	0.143
△SBP6, mmHg	-22.5 (-29.6,-15.3)	-15.2(-20.4,-10.0)	**0.022**
△DBP2, mmHg	-11.9(-15.9,-8.0)	-10.8(-13.8,-7.9)	0.498
△DBP4, mmHg	-12.9(-17.4,-8.5)	-9.5(-12.7,-6.2)	0.247
△DBP6, mmHg	-14.3(-18.9,-9.8)	-12.8(-16.1,-9.5)	0.346
△MAP2, mmHg	-15.3(-19.4,-11.3)	-11.6(-14.6,-8.5)	0.123
△MAP4, mmHg	-14.1(-18.7,-9.7)	-11,1(-14.4,-7.8)	0.141
△MAP6, mmHg	-17.1(-22.0,-12.1)	-13.6(-17.2,-10.0)	0.085

*P-values with the Bonferroni adjustment and BMI, gender, age adjustments were used in the model.

△SBP2, 4, 6 = SBP change from baseline at the end of 2, 4, and 6 weeks, respectively; △DBP2, △DBP4, and △DBP6 = DBP change from baseline at the end of 2, 4, and 6 weeks, respectively; △MAP2, △MAP4, and △MAP 6 = MAP change from baseline at the end of 2, 4, and 6 weeks, respectively.

Andrea Semplicini et al. reported that *RGS2* expression affects the response to antihypertensive treatment, and age is one of the key factors in regulating *RGS2* mRNA level [22]. Our stratification analyses also showed that the responses to α-ADR and β-ADR blocker treatment in patients with essential hypertension (stratified according to *RGS2* [-391, C>G] genotypes) were age- and genotype-dependent; these results are summarized in Table 3. However, no significant differences in the blood pressure responses were observed between the *RGS2* genetic polymorphisms in the calcium channel blocker (i.e., nitrendipine or azelnidipine) medication cohorts.

**Table 3:** Cardiovascular response to 4 weeks of α,β-ADR blocker therapy in EH patients, as stratified by the *RGS2* genotype.

Drugs	Genotype-Age (or) Gender-Specific	Variables	mean±S.D(mmHg)	P-value	*P-value
doxazosin	Men-GC/CC (16) v.s Women55y-GC/CC(9)	△SBP4	-17.4±14.3 v.s -0.1±15.2	0.020	0.049
		△DBP6	-13.1±7.6 v.s -3.2±11.1	0.007	0.023
		△MAP4	-16.1±7.1 v.s -6.0±8.8	0.012	0.005
		△MAP6	-14.6±8.1 v.s -0.3±12.9	0.004	0.009
bisoprolol or atenolol	Age⩽55y-GC/CC (16) v.s	△DBP4	-26.8±8.2 v.s -18.5±9.1	0.051	0.057
	Age>55y-GC/CC(7)				
doxazosin, bisoprolol atenolol	Age⩽55y-GG (22) v.s Age>55y-GG(19);	△HR4;	-13.8±14.9 v.s 5.5±10.6;	0.001	0.003
	Age⩽55y-GC/CC (35) v.s	△DBP4	-18.9±10.4 v.s -11.1±9.8	0.006	0.014
	Age>55y-GC/CC(38)	△MAP4	-20.1±10.0 v.s -12.7±10.2	0.017	0.019
doxazosin, celiprolol, bisoprolol atenolol	Age⩽55y-GG (27) v.s Age>55y-GG(30);	△HR4;	-11.1±14.7 v.s 4.3±9.5;	0.001	0.004
	Age⩽55y-GC/CC (53)	△DBP4	-16.9±9.6 v.s -11.5±8.9	0.011	0.008
	Age>55y-GC/CC(58)	△MAP4	-18.0±9.5 v.s v.s -13.3±9.1	0.058	0.012

P-values were appropriately adjusted with the Bonferroni corrections. BMI, gender, and age were adjusted in the model.

*P-values represent genotype and age interaction. △HR4 = the heart rate change from baseline at the end of 4 weeks, △DBP4 = the diastolic pressure change from baseline at the end of 4 weeks, △MAP4 = the mean arterial pressure change from baseline at the end of 4 weeks, △DBP6 = the diastolic pressure change from baseline at the end of 6 weeks, △MAP6 = the mean arterial pressure change from baseline at the end of 6 weeks.

## Discussion

Our study indicates that the hypotensive effects of ACE inhibitor (imidapril) or ARB (candesartan, irbesartan) therapy were more sensitive in the *RGS2* (-391 C>G) GG genotype carriers compared with the GC/CC genotype carriers, but no significant differences between these groups were observed in the responses to nitrendipine or azelnidipine administration. One previous study found that *RGS2* expression was markedly lower in hypertensive patients than normotensive individuals, and low *RGS2* expression was related to the increased ANGII-stimulated release of intracellular Ca^2+^ peaks in fibroblasts [2]. These data may support the premise that the *RGS2* (-391 C>G) genetic polymorphism results were related to the ACE inhibitor treatment responses rather than the CCB blockers. The ANG II-stimulated pathway was significantly associated with the reduction in *RGS2* expression and the lower concentration of intracellular Ca^2+^. Sugimoto et al.[23] reported that the promoter polymorphism of *RGS2* (-638, A>G) was related to the hypotensive effect of azelnidipine treatment at the end of eight weeks, but this mutation did not affect the *RGS2* expression. However, these results require further confirmation. Semplicini et al. demonstrated that *RGS2* expression was negatively correlated with a patient’s SBP response to antihypertensive medication [7]. One study found that *RGS2* mRNA levels increased because of the *RGS2* (-391 C>G) mutation [15]. The antihypertensive and organ protection effects of telmisartan or candesartan are greater in *RGS2* ^-/-^mice than in *RGS2* ^+/+^ mice [24,25]. Previous data support our results that the *RGS2* (-391 C>G) genetic variation was associated with responses to ACE inhibitors and ARB but not with CCB.

Unexpectedly, our analyses of the ACE inhibitor and ARB treatment groups showed that the SBP changes between the *RGS2* (-391 C>G) CC and CG/GG genotypes differed significantly at the end of 2 and 6 weeks (but not at the end of 4 weeks) after being treated with ARB (i.e., candesartan and irbesartan) medications (Table 2). However, current data do not indicate the mechanism underlying this phenomenon. Because of the lack of clinical data concerning the ACE inhibitor (imidapril) treatment at the end of 4 weeks in our study cohort, it is unknown whether a common mechanism is mediated by the Ang II-*RGS2* pathway. According to the literature, *RGS2* deletion promotes the sensitization of Ang II type1 receptors, and group VIA phospholipase A2 (iPLA2β) participates in the up-regulation of Ang II-induced *RGS2* mRNA and protein levels in vascular smooth muscle cells [26,27]. Ang II modulated *RGS2* transcription in a time- and concentration-dependent manner. Short time periods and low concentrations of Ang II-promoted *RGS2* expression; conversely, long time periods and high concentrations inhibited *RGS2* expression. Valsartan can reverse the reduced *RGS2* transcription [28]. These findings may help to improve our understanding of this phenomenon.

However, our data showed that the *RGS2* (-391 C>G) genetic variation was unrelated to the patients’ blood pressure responses after ACE inhibitor (imidapril) monotherapy. Ang-(1–7) can elevate blood pressure by promoting pro-inflammatory and pro-thrombotic effects. Moreover, Ang IV can activate AT4R and may contribute to cardiac damage [20,29]. Likewise, Ang IV can counteract the presence of AT2 receptors by increasing blood pressure and reducing renal blood flow in mice. Ang IV can ameliorate AngII-induced cardiac injury via AT4R and protect against acute cerebral ischemia via Ang IV-AT4R and NO-dependent mechanisms [21,30]. Therefore, this result may be attributed to the intricate mechanisms and the small sample size, while ARBs do not directly affect the action of Ang-(1–7).

Table 3 indicates that the interaction between the *RGS2* (-391 C>G) genetic polymorphism and the response to α,β-ADR blocker therapy was age-specific. Between age groups, the significant difference in HR from baseline was limited to the patients with the *RGS2* (-391 C>G) GG genotype. However, the MAP and DBP changes from baseline only occurred in the patients with the GC/CC genotype. Recent studies indicate that the α1-ADR and β-ADR agonists up-regulate *RGS2* mRNA levels through a cAMP-dependent pathway [8,10,12,31], and the *RGS2* (-391 C>G) mutation is associated with enhanced *RGS2* expression *in vitro* [15]. However, no underlying mechanisms have confirmed that the differential response to α,β-ADR blocker treatments between the *RGS2* (-391 C>G) genotype groups are age-dependent, and the expression of *RGS2* may change with age[22].

In conclusion, the results of the present study suggest that the *RGS2* (-391 C>G) polymorphism correlates to the responsiveness of antihypertensive drug therapy in Chinese EH patients, and *RGS2* (-391 C>G) may be a useful biomarker in determining the combination of certain antihypertensive drug therapies. Our results are limited by the heterogeneous group of drugs and the relatively small number of patients in each treatment arm. A separate replication cohort is needed to validate our findings.
